# Stab Incision Glaucoma Surgery: A Modified Guarded Filtration Procedure for Primary Open Angle Glaucoma

**DOI:** 10.1155/2016/2837562

**Published:** 2016-04-10

**Authors:** Soosan Jacob, Michele Figus, Dhivya Ashok Kumar, Ashvin Agarwal, Amar Agarwal, Saijimol Areeckal Incy

**Affiliations:** ^1^Dr. Agarwal's Eye Hospital and Eye Research Centre, 19 Cathedral Road, Chennai 600 086, India; ^2^University of Pisa, 56126 Pisa, Italy

## Abstract

*Purpose*. To describe a modified guarded filtration surgery, stab incision glaucoma surgery (SIGS), for primary open angle glaucoma (POAG).* Methods*. This prospective, interventional case series included patients with POAG (IOP ≥21 mmHg with glaucomatous visual field defects). After sliding superior conjunctiva down over limbus, 2.8 mm bevel-up keratome was used to create conjunctival entry and superficial corneoscleral tunnel in a single step starting 1.5 mm behind limbus. Lamellar corneoscleral tunnel was carefully dissected 0.5–1 mm into cornea and anterior chamber (AC) was entered. Kelly Descemet's punch (1 mm) was slid along the tunnel into AC to punch internal lip of the tunnel, thereby compromising it. Patency of ostium was assessed by injecting fluid in AC and visualizing leakage from tunnel. Conjunctival incision alone was sutured.* Results*. Mean preoperative IOP was 27.41 ± 5.54 mmHg and mean postoperative IOP was 16.47 ± 4.81 mmHg (*n* = 17). Mean reduction in IOP was 38.81 ± 16.55%. There was significant reduction of IOP (*p* < 0.000). 64.7% had IOP at final follow-up of <18 mmHg without medication and 82.35% had IOP <18 mmHg with ≤2 medications. No sight threatening complications were encountered.* Conclusion*. Satisfactory IOP control was noted after SIGS in interim follow-up (14.18 ± 1.88 months).

## 1. Introduction

Conjunctival dissection is an important step in glaucoma filtering surgery [1, 2]. Decreasing the intraoperative conjunctival manipulation may be expected to lead to less subconjunctival fibrosis and better aqueous drainage in the long term [3–5]. An accepted method of maintaining aqueous drainage has been conventional trabeculectomy where, after raising a conjunctival flap, an artificial channel is made between the anterior chamber and the subconjunctival space by means of a scleral flap [1]. Reduction of intraocular pressure (IOP) has been known to be sustained by this channel over a period [6–9]. In this paper, we have presented a modified technique of trabeculectomy for primary open angle glaucoma (POAG) where a scleral tunnel has been used with decreased conjunctival dissection. This technique was described by one of us (SJ).

## 2. Materials and Methods

This prospective interventional case series was carried out at Dr. Agarwal's Eye Hospital and Eye Research Centre, Chennai, India. Institutional review board (IRB) approval was obtained and the procedure conformed to declaration of Helsinki. Informed consent was obtained from all patients. Preoperative visual acuity was measured with Snellen acuity charts, IOP by Goldmann applanation tonometer, and anterior chamber depth by optical coherence tomography. Patients with primary open angle glaucoma of age 40 to 70 years, IOP more than 21 mmHg, and visual field defects on automated perimetry and who were willing for follow-up were included after informed consent. Patients with previous uveitis, conjunctival scarring, angle closure glaucoma, previous trabeculectomy, and poor visual acuity in fellow eye and one eyed patients were excluded. The follow-up visits were on day 1, day 7, 1 month, 3 months, 6 months, and so on.

A complete success was defined as an intraocular pressure of <18 mmHg without medications and qualified success was defined as reduction in IOP to <18 mmHg with two or less medications. Failure was defined as need for more than 2 medicines postoperatively for IOP control. Intraoperative or postsurgical event which required conversion to conventional trabeculectomy or resurgery to decrease IOP was also considered as failure. Any ocular adverse effect which required surgical or medical intervention for management apart from regular postoperative regimen was considered as complication of the surgery.

**Figure 1:**
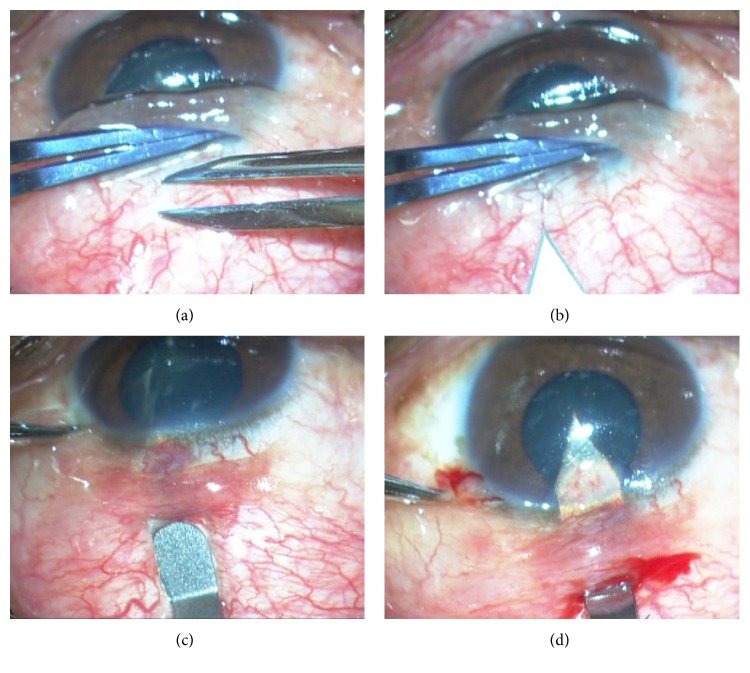
Stab incision glaucoma surgery (SIGS). (a), (b) Superior conjunctiva is slid downwards over the cornea and a 2.8 mm bevel-up metal keratome is introduced 1.5 mm behind the limbus. (c) Keratome is passed through the conjunctiva and into lamellar sclera and a superficial lamellar scleral tunnel is then dissected. (d) Keratome is introduced about 0.5–1 mm into the clear cornea and the anterior chamber (AC) is then entered.

**Figure 2:**
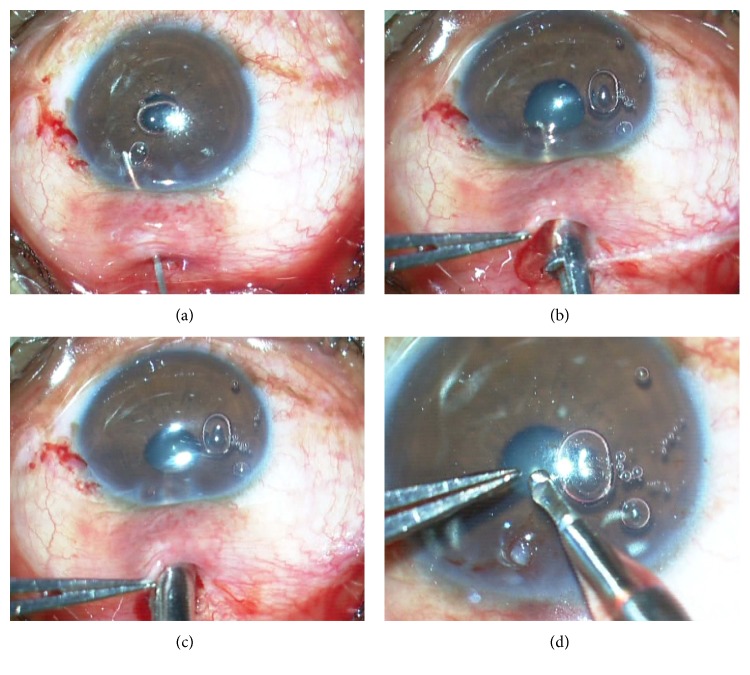
(a) Viscoelastic is injected into the AC. (b), (c) Kelly Descemet's punch used to punch the internal lip of the cornea. (d) The punched tissue from the posterior lip of cornea is seen.

**Figure 3:**
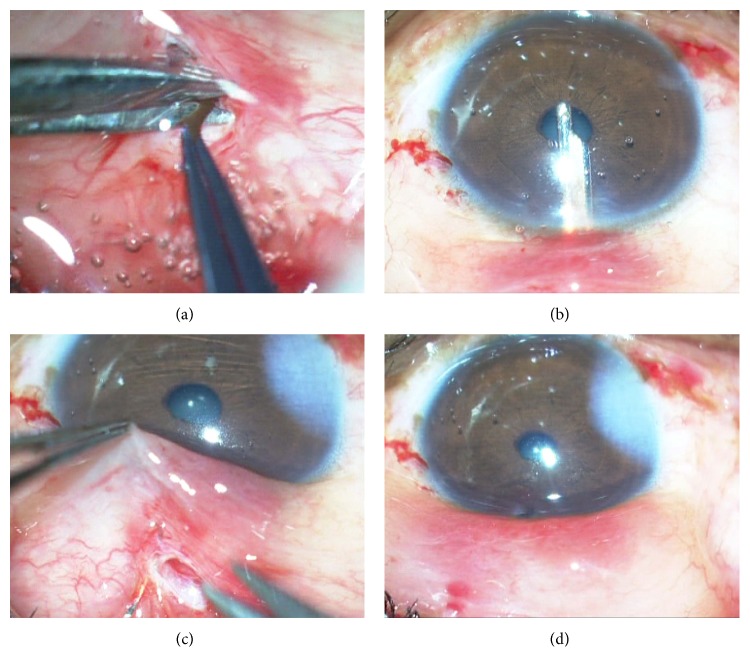
(a) A peripheral iridectomy (PI) is done with curved Vannas scissors by retracting basal iris through the tunnel with nontoothed forceps while the assistant retracts the conjunctiva for visualization. (b) Viscoelastic is washed out. (c) Patency of the tunnel is checked by irrigating the anterior chamber with balanced salt solution and looking for bleb formation. The conjunctival incision is then sutured with a running 10-0 nylon suture. (d) The bleb is seen lifted after side port irrigation.

Preoperatively, 0.2 mL of 0.01% mitomycin C (MMC) was injected subconjunctivally in the area of intended bleb creation. Peribulbar anesthesia was given and the eye was prepared and draped. The speculum was loosened slightly to allow more mobile conjunctiva and to prevent it from being pulled into the fornices. Superior conjunctiva was slid downwards over the cornea and a 2.8 mm bevel-up metal keratome was introduced 1.5 mm behind the limbus (Figures 1(a) and 1(b)). The tip of the keratome was passed through the conjunctiva into superficial lamellar sclera (Figure 1(c)). A superficial lamellar scleral tunnel was then dissected with side to side movements of the keratome till the limbus, keeping the depth of dissection such that the blade could be visualized through the overlying sclera and conjunctiva. At the limbus, the blade was angulated more superficially to correspond to the steeper corneal curvature and depth was confirmed by a dimpling of the cornea. The blade was then introduced 0.5 to 1 mm into clear cornea before entering the anterior chamber (AC) in a horizontal plane (Figure 1(d)). Downwards pressure on the keratome was avoided while entering the AC. The blade was then withdrawn and viscoelastic instilled into the AC through either a paracentesis or the stab incision glaucoma surgery (SIGS) tunnel entry (Figure 2(a)). The 1 mm Kelly Descemet's punch (Appasamy Associates, India) was then slid along the tunnel into the AC and with the punch facing downwards, the internal lip of the corneal section was punched (Figures 2(b) and 2(c)). After pushing the iris away with viscoelastic, the ostium thus initiated was extended posteriorly till the limbus with additional punches under visualization through the overlying clear cornea (Figure 2(d)). A peripheral iridectomy (PI) was done with curved Vannas scissors by retracting basal iris through the tunnel with nontoothed forceps while the assistant retracted the conjunctiva for visualization (Figure 3(a)). The iris was gently pushed back into the AC and a simcoe cannula inserted through the tunnel was used to wash out viscoelastic (Figure 3(b)). Balanced salt solution (BSS) was irrigated through the side port and leakage from the SIGS tunnel was assessed. End point looked for was a free flow of fluid on irrigation. Additional punches were taken in case of inadequate leak. The 2.8 mm conjunctival cut was sutured with a running 10-0 nylon suture (Figure 3(c)). BSS was injected through the side port to balloon the bleb (Figure 3(d)). Subconjunctival garamycin 0.5 mL and dexamethasone 0.5 mL were given in the inferior forniceal conjunctiva (Video file, at Supplementary Material available online at http://dx.doi.org/10.1155/2016/2837562).

### 2.1. Statistical Analysis

Data was entered in a Microsoft Excel Sheet (Microsoft Corp., Redmond, Washington, USA) and was analyzed using SPSS version 16.1 (SPSS Inc., Chicago, Illinois, USA). Continuous variables were expressed as means (±standard deviations) and categorical variables were expressed as individual counts. After testing for normality distribution of data, the statistical tests were allotted. Nonparametric tests were used for comparison. Differences were considered statistically significant when the *p* value was less than 0.05.

## 3. Results and Discussion

### 3.1. Results

Seventeen eyes of 17 patients (7 males, 10 females) underwent SIGS with preoperative subconjunctival MMC. The mean age was 57 ± 11.9 years. The mean follow-up was 14.18 ± 1.88 months. The mean preoperative IOP was 27.41 ± 5.54 mmHg (range 21–39 mm Hg) and the mean postoperative IOP was 16.47 ± 4.81 mmHg (range 9–28 mmHg). There was significant reduction in IOP from preoperative values (Wilcoxon signed rank test, *p* < 0.000). The mean reduction in IOP was 38.81 ± 16.55%. Preoperatively 7 out of 17 (41.1%) had advanced glaucomatous field loss and 10 out of 17 (58.8%) had moderate glaucomatous field loss. There was no significant change in pattern standard deviation (PSD) in visual field test (*p* = 0.068) from preoperative to postoperative period. The mean preoperative and postoperative PSD were 6.9 ± 2.7 Decibels and 6.97 ± 2.7 Decibels. The number of topical medications was reduced from a mean of 1.35 preoperatively to 0.59 postoperatively. There was significant reduction in number of medications used from pre- to postoperative period (*p* = 0.025, Wilcoxon signed rank test). A complete success defined as an intraocular pressure of <18 mmHg without medications was seen in 64.70% and 82.35% of patients maintaining an IOP of <18 mmHg with ≤2 medications. Preoperative corrected distance visual acuity (CDVA) measured by Snellen's distant visual acuity charts was 0.80 ± 0.28 and postoperative CDVA was 0.76 ± 0.25 (Wilcoxon signed rank test, *p* = 0.230). Intraoperative complications encountered were premature entry (*n* = 1; small basal punch taken), trapdoor hinging of internal corneal lip (*n* = 1; hinged lip excised), conjunctival buttonhole (*n* = 1; small, no intervention), Descemet's detachment (*n* = 1; small, managed with air bubble), and nonbasal PI (*n* = 1; no intervention). Postoperative complications encountered were microhyphema (*n* = 2; medical management), more than grade 2 AC reaction (*n* = 1; medical management), hypotony (*n* = 1, managed successfully with transconjunctival tunnel compression suture), and uncontrolled IOP (*n* = 6; 3 were managed medically and 3 underwent repeat surgery). There were no sight threatening complications such as bleb inflammation/infection or cystoid macular edema seen in any of the eyes.

### 3.2. Discussion

Traditional trabeculectomy has been a widely adopted procedure of choice [1, 2]. However, a common cause of glaucoma surgery failure is subconjunctival fibrosis of the bleb. Wound-healing response after filtering surgery can present in one of two ways, either as subconjunctival fibrosis or as sub-Tenon's encapsulation leading to lack of filtration and subsequent increase in IOP. In an attempt to reduce failure due to fibrosis, antimetabolites have been used; however, fibrosis and bleb failure are still reported [10]. Reducing excessive intraoperative manipulation of conjunctiva and Tenon's capsule has been shown to prevent fibrosis [7–13]. Nontraumatic subconjunctival dissection has also been tried to prevent inadvertent conjunctival handling [11–13].

Increased conjunctival manipulation can increase chances of buttonhole, tear, and subsequent fibrosis. A reduction in manipulation would help in reducing the same. Theoretically, positioning the conjunctival incision and scleral entry apart from each other (about 4 mm) may help in preventing direct conduit for microbes. Though there is no exact data on how much the incisions should be spaced for a significant protective effect, there was no inflammation or infection seen in our cases. However, similar to trabeculectomy, in the presence of a leak or wound dehiscence, this potential advantage may be lost and such situations should be avoided. Sliding the conjunctiva towards the limbus to avoid the conjunctival and scleral wounds from overlapping also decreases the risk of fibrosis between overlapping incisions. Mechanical pressure exerted by the conjunctival sutures is transmitted away from the scleral wound and there is therefore less contact zone between the 2 incisions.

SIGS reduces conjunctival dissection as the keratome produces a smooth linear entry. Subconjunctival drainage channels are therefore preserved intact to a larger extent as compared to trabeculectomy, thus potentially inducing less subconjunctival fibrosis and allowing better subconjunctival drainage. Bleb elevation is by hydrostatic expansion of largely intact subconjunctival tissue. In our experience, the creation of the tunnel was easy and one step and thereby surgery was easier and faster. Larger area of intact conjunctiva also has additional advantage of allowing greater space for a second glaucoma surgery if required. The single step tunnel approach in SIGS offers advantages of posteriorly directed aqueous flow as compared to leakage under a flap where flow occurs to three sides. This can prevent complications such as overhanging blebs and bleb dysesthesia. Once the learning curve is crossed, other potential advantages of this technique include ease of creation, less manipulation compared to a scleral flap, less likelihood of flap tears and buttonholes, and less suture related inflammation. In the extreme event of an expulsive hemorrhage, it can be rapidly closed.

Tunnel trabeculectomies that use a scleral tunnel have been described in the past as a successful mode of filtration surgery [14–16]. These involve creating a triplanar scleral tunnel after raising a conjunctival flap. SIGS follows this principle of tunnel trabeculectomy. However, it differs from these in the absence of conjunctival flap creation and in utilizing a biplanar scleral tunnel. The entry in SIGS is one step and conjunctival incisions as well as subconjunctival dissection are therefore minimized. A biplanar incision is a more advantageous geometry than a triplanar incision for better aqueous leakage postoperatively.

Significant intraoperative complications encountered were premature entry and trapdoor hinging of the posterior corneal lip. Premature entry occurred when the keratome pass was deep and entered the AC directly without a corneal component. This was managed by taking a single small punch which was sufficient to attain adequate leakage. A trapdoor hinging occurred when the corneal tunnel was deep and the posterior corneal lip gave way. This was managed by excising the hinge with Vannas scissors and then proceeding as usual.

**Figure 4:**
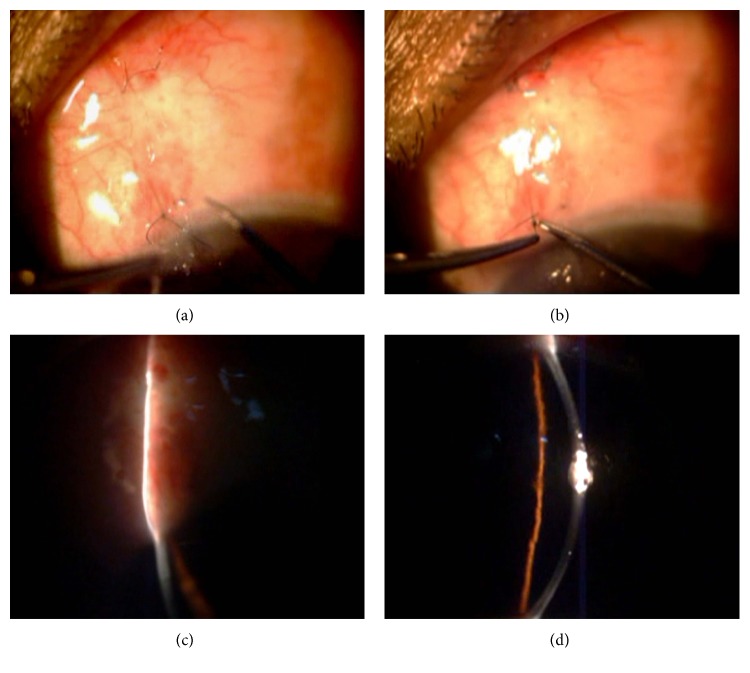
(a) Compression suture seen passed transconjunctivally. (b) Suture being removed after 3 weeks. (c) Shallow bleb seen formed after suture removal and mild massage. (d) Well-formed AC and controlled IOP at 15 mmHg.

Success rates after trabeculectomy with target pressure between 18 and 22 mmHg have been variously reported to range from 43% to 86% without medications [17–26] and from 59% to 98% with medications [17, 21, 22]. In our study, postoperatively, a complete success was possible in 64.7% with an IOP at final follow-up of <18 mmHg without medication and 82.35% with IOP <18 mmHg with two or less medications. Three patients had to undergo repeat surgery. One patient who had postoperative hypotony was managed by transconjunctival compression suture that compressed the tunnel and decreased flow. Transconjunctival suturing was easy to perform as the scleral tunnel was visible through overlying conjunctiva on stretching conjunctiva out over the tunnel area. The conjunctiva did not need to be cut for tunnel exposure to apply the suture and the suture was removed after 3 weeks (Figures 4(a)–4(d)). Other complications encountered were minor.

Though we did not put releasable suture in any patient in this case series, it is possible to do postoperative IOP management using laser suture lysis or removable suture technique in the same manner as is done in trabeculectomy [27]. This can be done by taking a releasable suture in the standard way through the tunnel and releasing it in the postoperative period. Unlike in trabeculectomy where suturing is possible under direct visualization, in SIGS visibility is comparatively more difficult. However, in our experience, the scleral lip of the tunnel is generally visible on pulling the conjunctival incision towards the limbus and this can make application of the releasable suture across the tunnel possible.

**Figure 5:**
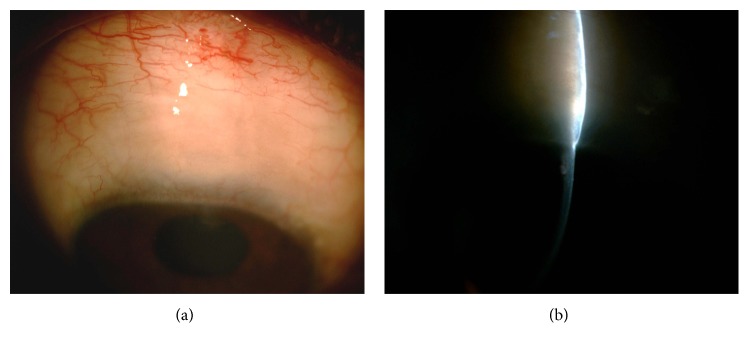
(a), (b) Slit lamp view showing diffuse, low bleb after SIGS.

**Figure 6:**
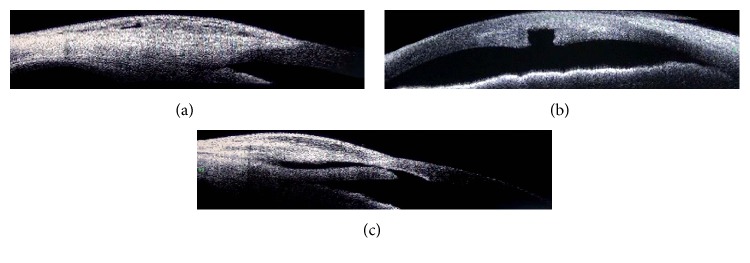
Anterior segment optical coherence tomography (ASOCT) showing bleb with multiple subconjunctival cystic spaces (a), corneal punch (b), and postoperative scleral tunnel and subscleral filtration path (c) at 10 months.

A potential disadvantage for this technique is that since all maneuvers are done under the tunnel, the technique appears partially blinded. However, we were able to visualize the scleral tunnel entrance under the conjunctiva, passage of the keratome, the corneal entry lip, corneal punch, and final diffuse bleb formation in all cases. Another potential disadvantage is the possibility of subconjunctival bleeding as cautery is not applied. In our experience, avoiding visible conjunctival and scleral blood vessels was generally enough to avoid large subconjunctival hemorrhage. Small oozing that occurred was not significant enough to interfere with surgery. However, patients on oral antiplatelet agents were advised to stop these medications for sufficient duration of time before surgery. The scleral tunnel incision though hidden underneath the conjunctiva could be visualized in all cases by withdrawing the conjunctival incision towards the limbus. Though only a gross assessment of flow was possible, in our experience, free flow of fluid and good ballooning of the bleb on side port irrigation when seen with a soft, yet stable AC lead to better outcomes (Figures 5(a) and 5(b); Figures 6(a)–6(c)).

## 4. Conclusion

Though long term results are still under evaluation, satisfactory IOP control was noted by us after SIGS in the interim follow-up. The small sample size and the limited follow-up make it difficult to draw a strong conclusion pertaining to long term outcomes and complication rate. This pilot study however serves to lay the foundation for further detailed analysis of this technique. A long term study in a larger population will be required to confirm the anatomical and functional outcomes of this modified technique.

## Supplementary Material

Superior conjunctiva is slid downwards over the cornea and a 2.8 mm bevel-up metal keratome is introduced 1.5 mm behind the limbus through the conjunctiva into superficial lamellar sclera to create a superficial scleral tunnel at a depth such that the blade is visualized through overlying sclera and conjunctiva. At the limbus, the blade is angulated more superficially to correspond to steeper corneal curvature and depth confirmed by a dimpling of the cornea. A 0.5 to 1 mm clear corneal tunnel is created and the AC entered in a horizontal plane. Downwards pressure on keratome is avoided while entering the AC. Viscoelastic is instilled into the AC and a 1 mm Kelly's Descemet's punch is used to punch the internal lip of the corneal section to create an ostium that is then extended posteriorly till the limbus. A peripheral iridectomy (PI) is done and the iris is then gently pushed back into the AC. A Simcoe cannula is inserted through the tunnel to wash out viscoelastic. Balanced salt solution is irrigated through the side port and leakage from the SIGS tunnel is assessed. End point looked for was a free flow of fluid on irrigation. Additional punches are taken in case of inadequate leak. The 2.8 mm conjunctival cut is sutured with a running 10-0 nylon suture. Additional BSS is injected through the side port to balloon the bleb.

## Additional Points

Free paper on this technique presented at American Society of Cataract and Refractive Surgery 2014 at Boston won the best paper award. Video film on this technique also won film festival award at American Society of Cataract and Refractive Surgery 2014 at Boston.
